# Comparison of Biportal and Uniportal Spine Endoscopy for Effectiveness and Perioperative Safety: A Systematic Review and Meta-Analysis

**DOI:** 10.7759/cureus.98764

**Published:** 2025-12-08

**Authors:** Suyash Singh, Purushottam Kumar, Priyanka Priyanka, Sumanth Masina, Satya D Pandey, Kurvatteppa Halemani

**Affiliations:** 1 Neurosurgery, All India Institute of Medical Sciences, Raebareli, IND; 2 Microbiology, All India Institute of Medical Sciences, Raebareli, IND; 3 Neurosurgery, Moti Lal Nehru Medical College, Prayagraj, IND; 4 Nursing, Sanjay Gandhi Postgraduate Institute of Medical Sciences, Lucknow, IND

**Keywords:** biportal endoscopic spine surgery, endoscopic decompression, lumbar decompression, minimally invasive spine surgery, uniportal endoscopic spine surgery

## Abstract

This study aimed to evaluate clinical effectiveness, surgical metrics (operative time and blood loss), postoperative recovery (hospital stay), patient-reported success (MacNab criteria), and complications between uniportal and biportal endoscopic spine surgery in patients with lumbar degenerative disease.

The systematic review and meta-analysis implemented Preferred Reporting Items for Systematic Reviews and Meta-Analyses and Cochrane guidelines for its methodology. The search of multiple databases during the last 20 years resulted in 53 studies that met the criteria for comparing biportal and uniportal endoscopic surgery approaches. The research team conducted data extraction and quality assessment, followed by random-effects proportion-based meta-analysis to present findings through tables and forest plots and risk-of-bias analysis. Heterogeneity was assessed using the I² statistic and τ² estimates.

The MacNab scores from the pooled analysis revealed no substantial difference between uniportal and biportal groups (pooled log odds ratio = -0.243, 95% CI = -1.02 to 0.53, p = 0.62), which demonstrated similar patient satisfaction and functional recovery between the two groups. Complication rates were also similar between the two approaches, with low heterogeneity across studies. The random-effects model for Visual Analog Scale leg pain in biportal surgery showed a significant decrease (intercept = 5.36, τ² = 5.40, I² = 98.3%, p < 0.001), which indicates that there was considerable variation and a general improvement in pain results. Risk-of-bias evaluation showed that most studies had low to moderate risk, while missing outcome data and outcome measurement domains had the lowest bias levels.

The two approaches of uniportal and biportal endoscopy deliver equal effectiveness and safety when performing lumbar spine decompression surgery. The biportal technique delivers superior postoperative pain management, but research indicates no major distinction in treatment results. Given the generally low to moderate risk of bias and consistency across studies, these findings support the use of either technique based on surgeon expertise and patient preference.

## Introduction and background

Spinal disorders create disability worldwide, and lumbar degenerative disease (LDD) stands as the main reason for this problem [1]. The condition includes degenerative disc disease, disc herniation, spinal stenosis, and degenerative spondylolisthesis, which cause chronic low back pain and radiculopathy, and neurogenic claudication that severely affects daily life [2,3]. The worldwide aging population will result in higher LDD rates and increased social expenses, according to projected data [1].

The first steps in treating LDD include physical therapy and medication, and interventional pain procedures [4]. The treatment plan includes surgical procedures for patients who fail to respond to treatment and experience worsening neurological symptoms [5]. Spinal surgery has undergone a fundamental transformation through minimally invasive methods, which work to deliver results matching or surpassing traditional methods by causing less damage to muscles and soft tissues and minimizing blood loss and speeding up recovery time [6,7].

Endoscopic spine surgery stands as a fundamental advancement in the field of minimally invasive spine care among these recent developments. The main advantage of this method enables the display of neural elements and their decompression while preserving the posterior spinal structures [8]. Full-endoscopic surgical methods include two main approaches: uniportal and biportal. The uniportal endoscopic technique requires only one entry point for both endoscope insertion and working instrument access, which may result in smaller incisions and less tissue damage [9]. The biportal endoscopic technique requires two separate entry points to achieve both endoscopic visualization and surgical tool insertion. The two layers separate to enhance surgical accessibility and improve irrigation flow, which enables better visualization and allows for more complex pathological procedures [10,11].

The medical field has demonstrated increasing interest in uniportal and biportal endoscopy, which has generated various studies to evaluate their operational safety and performance. Research findings have produced conflicting results, which prevent the identification of the most effective treatment approach. Multiple meta-analyses exist in the literature [9,12-20], but a new analysis is needed to combine current evidence with a strong research approach.

The researchers conducted a systematic review and meta-analysis to evaluate the clinical results and surgical data, and postoperative complications between uniportal and biportal endoscopic spine surgery for LDD. The evaluation of direct comparative research and secondary evidence will help clinicians to make evidence-based decisions for their surgical interventions.

## Review

Methods

Study Design and Registration

The Preferred Reporting Items for Systematic Reviews and Meta-Analyses (PRISMA) guidelines [21] and the Cochrane Handbook for Systematic Reviews of Interventions [22] were followed in conducting this systematic review and meta-analysis.

Objectives

The research objective focused on assessing uniportal versus biportal endoscopic spine surgery methods for treating LDD through synthesizing evidence of pain intensity and functional outcomes, and safety data. The study included two main objectives: to evaluate the effectiveness of the procedure and to compare intraoperative parameters (operative time, blood loss), postoperative recovery (hospital stay), patient-reported success (MacNab criteria), and complications.

Information Sources and Search Strategy

A systematic literature search was performed across multiple electronic databases, including PubMed, Embase, Cochrane Central Register of Controlled Trials, Web of Science, and China National Knowledge Infrastructure (for Chinese literature), from January 2005 to May 2025. A thorough mix of keywords and medical topic headings (Medical Subject Headings) associated with "uniportal endoscopy", "biportal endoscopy", "percutaneous endoscopy", "full endoscopy", "percutaneous interlaminar endoscopy", and "lumbar spine" was used in the search strategy. Initially, there were no language constraints; however, the final analysis only included research that was published in either Chinese or English.

Selection Criteria

We included randomized controlled trials (RCTs) and prospective or retrospective comparative studies that directly compared uniportal vs. biportal techniques or provided sufficient data (Visual Analog Scale (VAS) for back pain, VAS for leg pain, and Oswestry Disability Index (ODI)) for each technique separately. We excluded case reports, conference abstracts, and studies without extractable outcome data.

Study Selection and Data Extraction

**Figure 1:**
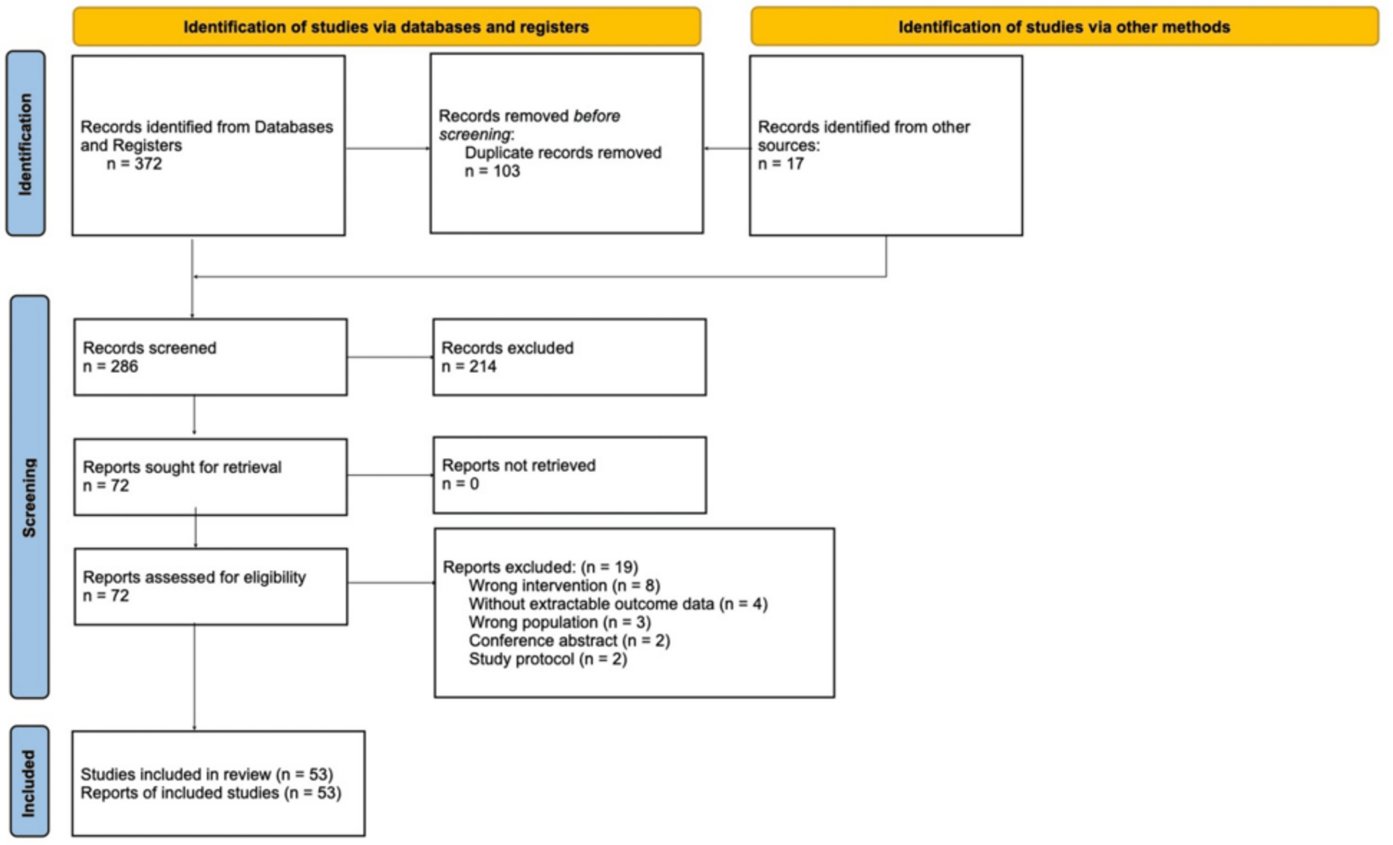
PRISMA diagram illustrating search strategy PRISMA: Preferred Reporting Items for Systematic reviews and Meta-Analyses

Two reviewers independently screened the titles/abstracts and subsequently reviewed the full-text articles against the eligibility criteria. A third reviewer was consulted or discussed to resolve disagreements. A PRISMA flow diagram (Figure 1) was used to record the study selection procedure.

Using a standardized data extraction form, two reviewers independently extracted the following information: first author, publication year, country, study design, sample size, patient demographics, surgical details, and outcome data (mean, standard deviation, and sample size for continuous outcomes; event counts for dichotomous outcomes).

Risk of Bias Assessment

**Figure 2:**
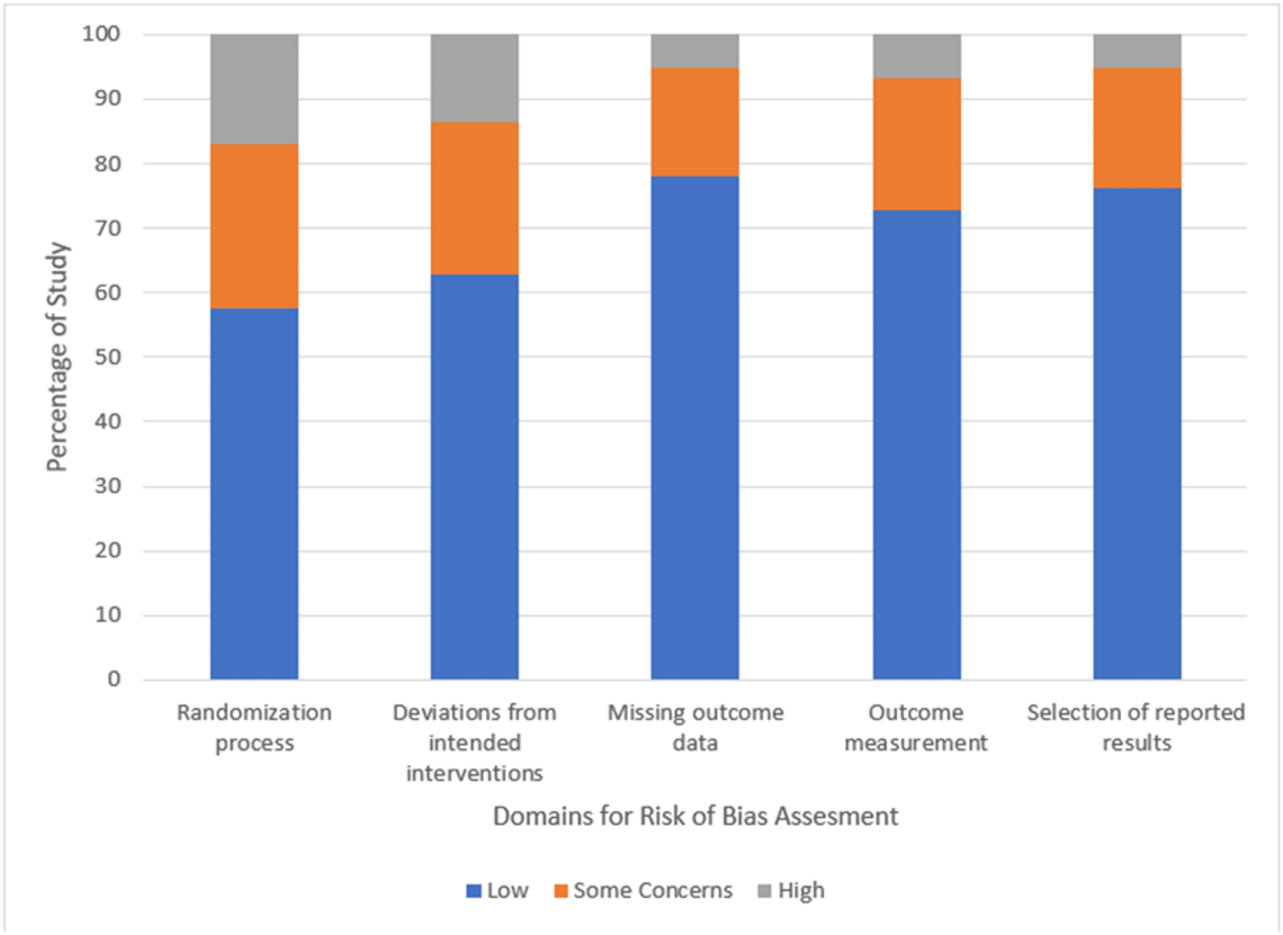
Risk of bias assessment summary (in percentage) Image credit: This is an original image created by the authors Purushottam Kumar and Suyash Singh

The Cochrane Risk of Bias (RoB) tool 2.0 was used to evaluate the included studies across five domains: randomization process, deviations from intended interventions, missing outcome data, outcome measurement, and selection of reported results [23]. Each domain was categorized as “high RoB,” “some concerns,” or “low RoB.” The overall RoB for each study was determined based on domain ratings and shown in Figure 2.

A study was considered “low risk” if all domains were rated as having low RoB. If one or more domains raised some concerns while the rest were low RoB, the overall rating was “some concerns.” Finally, a study was classified as “high risk” if at least one domain was rated as high or if multiple domains raised concerns. The Newcastle-Ottawa Scale was used for nonrandomized studies [24]. Assessments were conducted independently by two reviewers, and disagreements were resolved by consensus.

Quality-of-Evidence Assessment

Two independent reviewers used the Grading of Recommendations, Assessment, Development, and Evaluation method to assess the quality of evidence for each pooled analysis [25]. Consensus talks or senior reviewer (SS and SDP) consultations were used to settle disagreements. There were four categories for the quality of the evidence: good, moderate, low, and extremely low. Although RCT evidence is originally regarded as high-quality, it can be devalued due to publication bias, inconsistent outcomes, indirectness of evidence, imprecision, and RoB. On the other hand, if a significant impact was noted, there was a dose-response connection, or reasonable confounding may change the effect, the quality could be improved.

Outcomes

The primary outcomes included pain intensity (VAS for leg and back pain), functional status (ODI), and complication rates. The secondary outcomes included operative time, intraoperative blood loss, length of hospital stay, and patient-reported success rates (MacNab criteria).

Data Synthesis and Statistical Analysis

All statistical analyses were performed using R software (version 4.3.0; R Foundation for Statistical Computing, Vienna, Austria) with the metafor, meta, dmetar, bayesmeta, and ggplot2 packages. For continuous outcomes measured on different scales (VAS, ODI), the standardized mean difference (SMD) was calculated with 95% confidence intervals (CIs). For outcomes measured on identical scales (operative time in minutes, blood loss in milliliters, and hospital stay in days), the mean difference (MD) was calculated to enhance clinical interpretability. Dichotomous outcomes were analyzed using odds ratios (OR) with 95% CIs.

A random-effects model (DerSimonian-Laird method) was applied to all analyses owing to the anticipated clinical heterogeneity. Heterogeneity was quantified using the I² statistic, with I² > 50% considered to represent substantial heterogeneity. The between-study variance was estimated using tau². Sensitivity analyses were conducted using a leave-one-out analysis. Publication bias was evaluated visually using funnel plots and statistically using Egger's regression test when ≥10 studies were available for an outcome.

For complication rates, a Bayesian meta-analysis was conducted using the bayesmeta package with noninformative priors (normal distribution with mean = 0 and SD = 1 for the log OR and uniform prior for the between-study heterogeneity). Markov Chain Monte Carlo sampling was performed with 50,000 iterations with a 5,000-iteration burn-in period.

Results

Fifty-three papers [26-78] were included in the analysis and review, encompassing 4,556 patients and 4,713 cases, with an average follow-up duration of 12 months. The mean age of the population was 61.77 ± 10.28 years, with 50.24% being female patients. Surgery was conducted on 4,713 lumbar levels, with L4-5 being the most frequent level (58.19%).

Primary Outcome

VAS for leg pain: Pain intensity levels for the leg during the follow-up period were reported in 35 studies [26-60] evaluating the biportal approach, 24 studies [26,39,47,52-72] on the uniportal approach, and an additional 11 studies [26,47,52-60] comparing biportal vs. uniportal approaches.

Biportal approach: The SMDs ranged from 0.7940 to 11.3292, with all estimates being positive (100%). The combined average SMDs from the model were 5.3370 (95% CI = 4.5377-6.1363) (Table 1). Consequently, this outcome shows a statistically significant divergence from zero (z = 13.0869, p < 0.0001), indicating a notable enhancement in leg pain.

**Table 1:** Random-effects meta-analysis and heterogeneity statistics for VAS (leg pain) in the biportal approach (k = 35) Tau² estimator: restricted maximum-likelihood SE: standard error, CI: confidence interval, df: degrees of freedom, VAS: Visual Analog Scale

Parameter	Estimate	SE	Z	p value	95% CI		τ	τ² (SE)	I² (%)	H²	R²	df	Q	p (heterogeneity)
					Lower bound	Upper bound								
Intercept	5.34	0.408	13.1	<0.001	4.538	6.136	2.353	5.535 (1.4106)	98.37	61.342	-	34.000	1,971.499	<0.001

**Figure 3:**
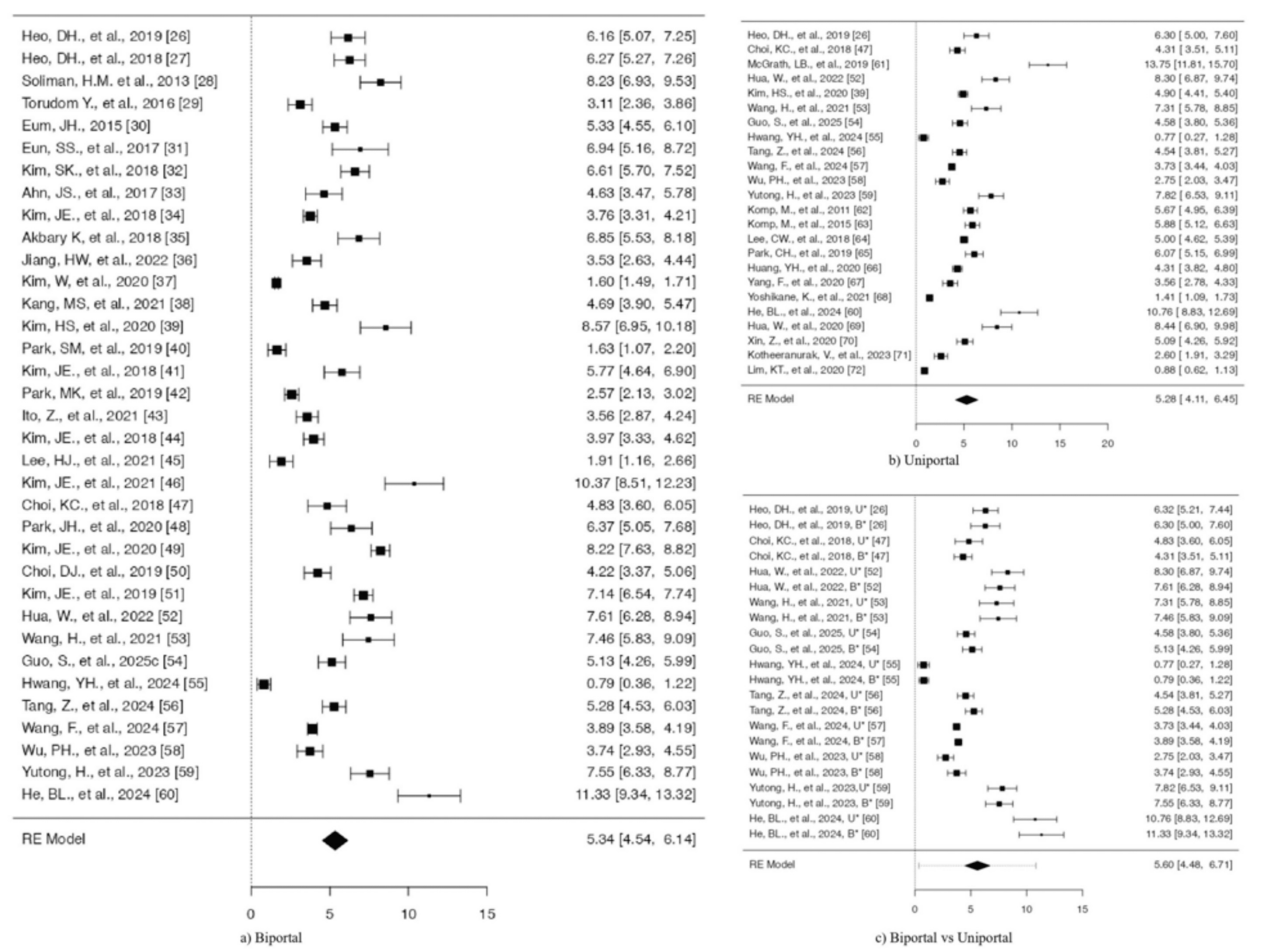
Forest plots comparing VAS for leg pain. Outcomes for (a) biportal endoscopic surgery and (b) uniportal endoscopic surgery. (c) Comparative analysis between biportal and uniportal approaches B*: Biportal approach, U*: Uniportal approach, VAS: Visual Analog Scale, RE: random effects

Based on the Q-test (displayed in Table 1), the actual results seem to be varied (Q(34) = 1,971.4989, p < 0.0001, tau² = 5.5350, I² = 98.3698%). A 95% prediction interval for the actual results ranges from 0.6571 to 10.0169. Therefore, despite some variations, the majority of individual study effects are likely to align with the direction of the pooled effect. The forest plot Figure 3a shows the SMDs and their CIs for the included studies. The analysis did not detect any studies that exceeded the threshold (± 3.1888) for outliers or showed excessive influence on the results. The publication bias tests produced significant results at p < 0.0001 and p < 0.0069.

Uniportal approach: The majority of estimations were positive (100%), and the observed SMDs ranged from 0.7748 to 13.7529, suggesting a steady improvement in leg discomfort. The average result is statistically significant (z = 8.8371, p < 0.0001), showing a strong overall impact in favor of the intervention. The estimated average SMDs based on the model were 5.2815 with (95% CI = 4.1101-6.4529) (Table 2).

**Table 2:** Random-effects meta-analysis and heterogeneity statistics for VAS (leg pain) in the uniportal approach (k = 24) Tau² estimator: restricted maximum-likelihood SE: standard error, CI: confidence interval, df: degrees of freedom, VAS: Visual Analog Scale

Parameter	Estimate	SE	Z	p value	95% CI		τ	τ² (SE)	I² (%)	H²	R²	df	Q	p (heterogeneity)
					Lower bound	Upper bound								
Intercept	5.28	0.598	8.84	-	4.110	6.453	2.883	8.3123 (2.5268)	98.95	94.985	-	23.000	1,235.243	<0.001

According to the Q-test, the true outcomes appear to be heterogeneous (Q(23) = 11,256.1248, p < 0.0001, tau² = 8.3123, I² = 98.9472%) (Table 2). A 95% prediction interval for the true outcomes is given by -0.4894 to 11.0524. Hence, although the average outcome is estimated to be positive, in some studies the true outcome may in fact be negative. The forest plot is shown in Figure 3b.

One research by McGrath et al. [61] has a value greater than ±3.0902, which may be an anomaly in the context of this model, according to an analysis of the studentized residuals. Potential asymmetry was revealed by the regression test and the rank correlation (p = 0.0013 and p < 0.0001, respectively).

Biportal vs. uniportal: The study comprised a total of k = 11 papers that contrasted uniportal and biportal techniques. The majority of estimations were positive (100%), and the observed SMDs varied from 0.7748 to 11.3292, suggesting a steady trend in favor of the biportal approach. The model yielded an estimated average SMD of 5.5952 (95% CI = 4.4774-6.7129). There was a significant difference in the average result (z = 9.8107, p < 0.0001). This implies that individuals treated with the biportal technique often reported better leg discomfort than those treated with the uniportal approach.

The true results seem to be diverse, according to the Q-test (Q(21) = 690.6922, p < 0.0001, tau² = 6.8232, I² = 98.1421%). However, the 95% prediction interval for the real outcomes, which ranges from 0.3549 to 10.8354, shows that the estimated average outcome and the true outcomes of the majority of the studies are largely in the same direction. Table 3 and Figure 3c show the model's overall result.

**Table 3:** Random-effects meta-analysis and heterogeneity statistics for VAS (leg pain) in uniportal vs. biportal approach Tau² estimator: restricted maximum-likelihood SE: standard error, CI: confidence interval, df: degrees of freedom, VAS: Visual Analog Scale

Parameter	Estimate	SE	Z	p value	95% CI		τ	τ² (SE)	I² (%)	H²	R²	df	Q	p (heterogeneity)
					Lower bound	Upper bound								
Intercept	5.60	0.570	9.81	-	4.477	6.713	2.612	6.8232 (2.2069)	98.14	53.823	-	21.000	690.692	<0.001

There was barely any indication of outliers in the context of this model because none of the studies had studentized residuals greater than ±3.0521. None of the studies could be deemed unduly influential based on Cook's distances. The regression test and the rank correlation both suggested possible asymmetry (p < 0.0001 and p < 0.0001, respectively).

VAS for back pain: The level of pain intensity for the back within the follow-up period is available for 28 articles [26-29,31,32,36,41-43,45-58,60,73-75] in the biportal approach, 22 articles [26,47,52-58,60-64,66-72,75] in the uniportal approach, and 11 articles [26,47,52-58,60,75] in the biportal vs. uniportal approach.

Biportal approach: Most estimations were positive (100%), and the observed SMDs varied from 0.7850 to 6.5172. The model yielded an estimated average SMD of 3.4982 (95% CI = 2.9820-4.0145) (Table 4). As a result, the average result was substantially different from zero (z = 13.2814, p < 0.0001).

**Table 4:** Random-effects meta-analysis and heterogeneity statistics for VAS (back pain) in the biportal approach Tau² estimator: restricted maximum-likelihood SE: standard error, CI: confidence interval, df: degrees of freedom, VAS: Visual Analog Scale

Parameter	Estimate	SE	Z	p value	95% CI		τ	τ² (SE)	I² (%)	H²	R²	df	Q	p (heterogeneity)
					Lower bound	Upper bound								
Intercept	3.50	0.263	13.3	-	2.982	4.014	1.334	1.7788 (0.5276)	95.24	20.996	-	27.000	436.344	<0.001

**Figure 4:**
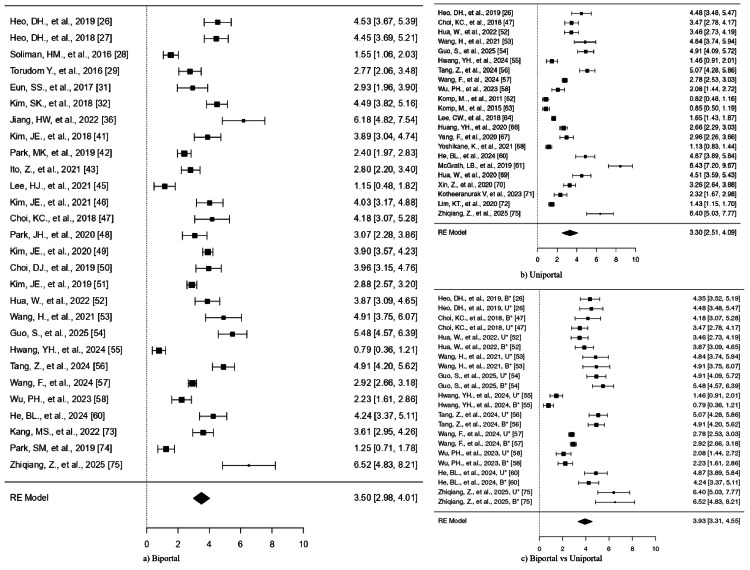
Forest plots comparing VAS for back pain. Outcomes for (a) biportal endoscopic surgery and (b) uniportal endoscopic surgery. (c) Comparative analysis between biportal and uniportal approaches B*: biportal approach, U*: uniportal approach, VAS: Visual Analog Scale, RE: random effects

The Q-test indicates that the real results seem to be diverse (Q(28) = 436.3342, p < 0.0001, tau² = 1.7788, I² = 95.2371%) (Table 4). The range of 0.8337-6.1628 is a 95% prediction interval for the actual results. Therefore, the genuine results of the studies are typically in the same direction as the predicted average outcome, notwithstanding the possibility of some variability. The forest plot of the SMD for the included study is shown in Figure 4a.

The analysis did not find any studies that exceeded the threshold (±3.1237) for outliers or showed excessive influence on the results. The publication bias tests produced significant results at p = 0.0015 and p < 0.0001.

Uniportal approach: The SMDs spanned between 0.8199 and 8.4348 while all studies reported positive results (100%). The model produced a pooled SMD of 3.2982 (95% CI = 2.5064-4.0899), which showed a statistically significant improvement (z = 8.1647, p < 0.0001) (see Table 5).

**Table 5:** Random-effects meta-analysis and heterogeneity statistics for VAS (back pain) in the uniportal approach Tau² estimator: restricted maximum-likelihood SE: standard error, CI: confidence interval, df: degrees of freedom, VAS: Visual Analog Scale

Parameter	Estimate	SE	Z	p value	95% CI		τ	τ² (SE)	I² (%)	H²	R²	df	Q	p (heterogeneity)
					Lower bound	Upper bound								
Intercept	3.30	0.404	8.15	<0.001	2.506	4.090	1.857	3.4479 (1.1073)	98.42	63.480	-	21.000	616.116	<0.001

The studies showed significant heterogeneity because the Q-test result was 616.1162 (p < 0.0001) and τ² was 3.4479, while I² reached 98.42%. The 95% prediction interval (−0.4263 to 7.0227) showed that studies shared a common direction of effect despite existing heterogeneity. The forest plot for this analysis appears in Figure 4b.

Studentized residual analysis revealed that McGrath et al. presented an outlier value that exceeded ±3.0521. The study results from Cook’s distance analysis suggested that this particular study could impact the overall results. The rank correlation test and regression test both showed evidence of publication bias (p = 0.0001 and p < 0.0001, respectively).

Biportal vs. uniportal: The research included 11 studies that compared biportal and uniportal surgical methods. The SMD values from the studies spanned between 0.7850 and 6.5172, while all studies reported positive results (100%). As shown in Table 6, the model produced a pooled SMD of 3.9335 (95% CI = 3.3141-4.5528), which confirmed the biportal approach performed better than the uniportal approach (z = 12.4476, p < 0.0001).

**Table 6:** Random-effects meta-analysis and heterogeneity statistics for VAS (back pain) in uniportal vs. biportal approach Tau² estimator: restricted maximum-likelihood SE: standard error, CI: confidence interval, df: degrees of freedom, VAS: Visual Analog Scale

Parameter	Estimate	SE	Z	p value	95% CI		τ	τ² (SE)	I² (%)	H²	R²	df	Q	p (heterogeneity)
					Lower bound	Upper bound								
Intercept	3.93	0.316	12.4	<0.001	3.314	4.553	1.414	1.9995 (0.6767)	95.33	21.410	-	21.000	366.335	<0.001

The model produced a pooled SMD of 3.9335 (95% CI = 3.3141-4.5528), which showed the biportal approach outperformed the uniportal approach (z = 12.4476, p < 0.0001).

The studies showed high variability (Q(21) = 366.3347, p < 0.0001; τ² = 1.9995; I² = 95.33%), yet the effects pointed in the same direction (prediction interval: 1.0936-6.7733). The forest plot appears in Figure 4c. The analysis did not detect any studies that exceeded the threshold for outliers or showed excessive influence on the results. The publication bias tests produced significant results at p = 0.0001 and p < 0.0001.

Oswestry disability index: The meta-analysis included 37 studies [26-32,34-47,49,51-60,62,63,75-77], which reported postoperative ODI scores for biportal surgery and 24 studies [26,47,52-67,69-72,75,78] for uniportal surgery and 12 studies [26,47,52-60,75] that compared both techniques.

**Figure 5:**
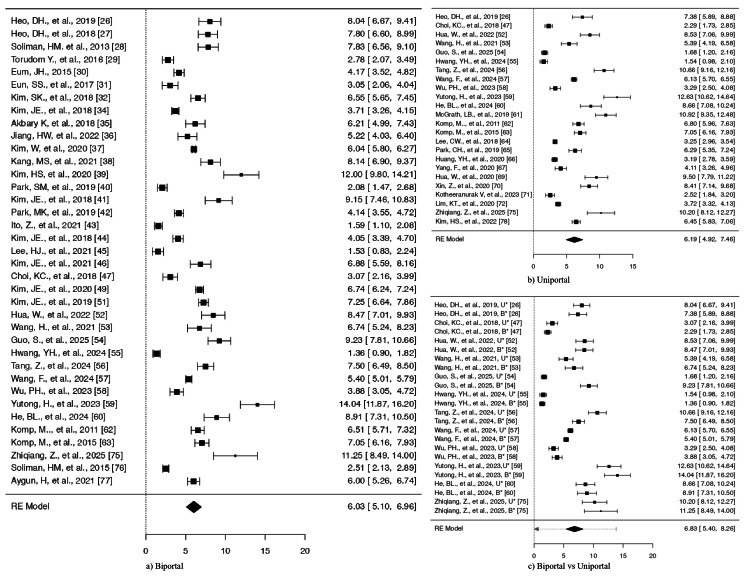
Forest plots comparing ODI. Outcomes for (a) biportal endoscopic surgery and (b) uniportal endoscopic surgery. (c) Comparative analysis between biportal and uniportal approaches B*: biportal approach, U*: uniportal approach, ODI: Oswestry Disability Index, RE: random effects

Biportal approach: The analysis included 37 studies that investigated the biportal approach. The SMD values spanned from 1.3599 to 14.0387 while all studies reported positive results (100%). The model produced a pooled SMD of 6.0293 (95% CI = 5.1650-6.9628), which demonstrated significant postoperative improvement (z = 12.6580, p < 0.0001) (Figure 5a and Table 7).

**Table 7:** Random-effects meta-analysis and heterogeneity statistics for ODI in the biportal approach Tau² estimator: restricted maximum-likelihood SE: standard error, CI: confidence interval, df: degrees of freedom, ODI: Oswestry Disability Index

Parameter	Estimate	SE	Z	p value	95% CI		τ	τ² (SE)	I² (%)	H²	R²	df	Q	p (heterogeneity)
					Lower bound	Upper bound								
Intercept	6.03	0.476	12.7	-	5.096	6.963	2.838	8.057 (1.977)	98.53	68.084	-	36.000	1,472.120	-

The studies demonstrated high levels of heterogeneity because Q(36) = 1,472.1197 (p < 0.0001) and τ² = 8.0570 and I² = 98.53%. The 95% prediction interval (0.3882-11.6704) showed that the studies demonstrated consistent effects, although they showed heterogeneity. The studentized residual test did not detect any studies that exceeded the threshold value of ±3.2048. The study by Yutong et al. [59] showed a moderate influence on the results. The funnel plot analysis revealed possible publication bias through its rank correlation test (p = 0.0010) and regression test (p < 0.0001).

Uniportal approach: The analysis included 24 studies, which reported SMD values between 1.5390 and 12.6297. The model produced a pooled SMD of 6.1902 (95% CI = 4.9195-7.4609), which showed a significant improvement (z = 9.5479, p < 0.0001) (Figure 5b and Table 8).

**Table 8:** Random-effects meta-analysis and heterogeneity statistics for ODI in the uniportal approach Tau² estimator: restricted maximum-likelihood SE: standard error, CI: confidence interval, df: degrees of freedom, ODI: Oswestry Disability Index

Parameter	Estimate	SE	Z	p value	95% CI		τ	τ² (SE)	I² (%)	H²	R²	df	Q	p (heterogeneity)
					Lower bound	Upper bound								
Intercept	6.19	0.648	9.55	-	4.919	7.461	3.123	9.7519 (2.9735)	98.81	83.734	-	23.000	968.425	-

The studies showed high levels of heterogeneity because Q(23) = 968.4250 (p < 0.0001) and τ² = 9.7519 and I² = 98.81%. The prediction interval (-0.0609 to 12.4413) showed that the effects pointed in the same direction despite varying magnitudes. The analysis did not find any studies that exceeded the threshold for outliers or showed excessive influence. The publication bias tests produced significant results at p = 0.0036 and p < 0.0001.

Biportal vs. uniportal: The research included 12 studies that directly compared both surgical methods. The SMD values from all studies were positive and ranged from 1.3599 to 14.0387. The model produced a pooled SMD of 6.8276 (95% CI = 5.3996-8.2555), which showed a significant difference between the two approaches (z = 9.3714, p < 0.0001) (Figure 5c and Table 9).

**Table 9:** Random-effects meta-analysis and heterogeneity statistics for ODI in uniportal vs. biportal approach Tau² estimator: restricted maximum-likelihood SE: standard error, CI: confidence interval, df: degrees of freedom, ODI: Oswestry Disability Index

Parameter	Estimate	SE	Z	p value	95% CI		τ	τ² (SE)	I² (%)	H²	R²	df	Q	p (heterogeneity)
					Lower bound	Upper bound								
Intercept	6.83	0.729	9.37	-	5.400	8.256	3.500	12.2498 (3.7546)	98.61	71.981	-	23.000	1,068.998	-

The studies demonstrated high levels of heterogeneity because Q(23) = 1,068.9979 (p < 0.0001) and τ² = 12.2498 and I² = 98.61%. The prediction interval (-0.1793 to 13.8344) showed that most studies supported the biportal approach, although the effect sizes might vary. The analysis did not detect any studies that exceeded the threshold for outliers or showed excessive influence. The funnel plot tests revealed asymmetry at p = 0.0082 and p < 0.0001.

Secondary Outcomes

**Figure 6:**
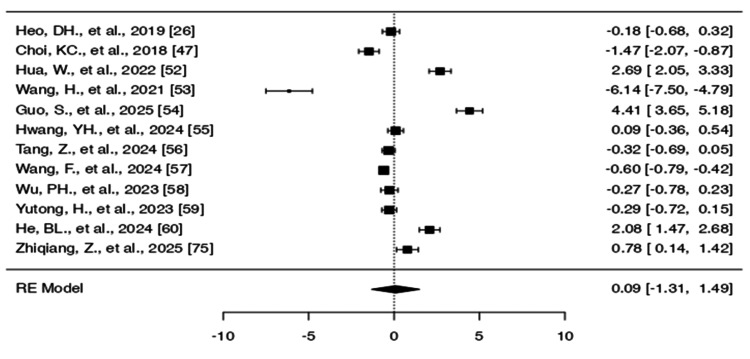
Forest plots comparing intraoperative time for biportal vs. uniportal approaches RE: random effects

Intraoperative time (biportal vs. uniportal): The article included 12 studies [26,47,52-60,75] that measured the duration of surgical operations. The SMD values spanned from 6.1425 to 4.4149, while 58% of the results showed negative values. The model produced a pooled SMD of 0.0893 (95% CI = 1.3140-1.4927), which failed to demonstrate any significant difference between the two approaches (z = 0.1248, p = 0.9007) (see Figure 6 and Table 10). The results showed significant differences between studies (Q(11) = 392.9611, p < 0.0001; τ² = 6.0449; I² = 99.10%) while the prediction interval (-4.9297 to 5.1084) showed extensive variation between studies. The study by Wang et al. showed signs of being an outlier that could affect the results. The analysis found no evidence of publication bias through rank correlation (p = 0.1160) or regression (p = 0.1708).

**Table 10:** Random-effects meta-analysis and heterogeneity statistics for operation time in uniportal vs. biportal approach Tau² estimator: restricted maximum-likelihood SE: standard error, CI: confidence interval, df: degrees of freedom

Parameter	Estimate	SE	Z	p value	95% CI		τ	τ² (SE)	I² (%)	H²	R²	df	Q	p (Heterogeneity)
					Lower bound	Upper bound								
Intercept	0.0893	0.716	0.125	0.901	-1.314	1.493	2.459	6.0449 (2.6228)	99.1	111.239	-	11.000	392.961	-

Intraoperative blood loss: The biportal approach received data from 11 studies [32,35,36,38,39,45,56-58,76,77] while seven studies [56-58,61,69-71] reported data for the uniportal approach and three studies [56-58] compared both approaches directly.

**Figure 7:**
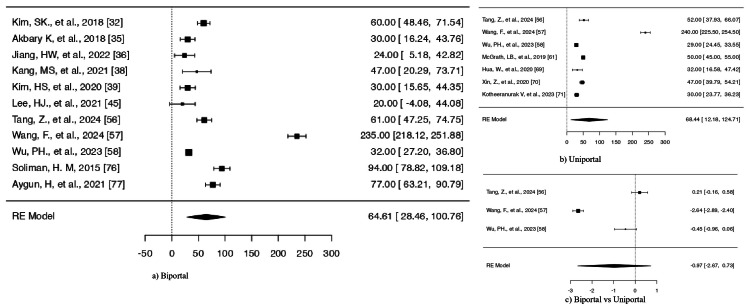
Forest plots intraoperative blood loss. Outcomes for (a) biportal endoscopic surgery and (b) uniportal endoscopic surgery. (c) Comparative analysis between biportal and uniportal approaches RE: random effects

Biportal: The combined blood loss amount from all studies reached 64.61 mL with a standard error of 18.4 and a 95% CI between 28.46 and 100.76. The overall effect proved statistically significant according to the results shown in Figure 7a and Table 11.

**Table 11:** Random-effects meta-analysis and heterogeneity statistics for intra-operative blood loss in the biportal approach Tau² estimator: restricted maximum-likelihood SE: standard error, CI: confidence interval, df: degrees of freedom

Parameter	Estimate	SE	Z	p value	95% CI		τ	τ² (SE)	I² (%)	H²	R²	df	Q	p (heterogeneity)
					Lower bound	Upper bound								
Intercept	64.6	18.4	3.50	-	28.456	100.761	60.581	3,670.0922 (1,673.6651)	98.85	86.856	-	10.000	596.514	-

The high level of heterogeneity (Q(10) = 596.514, p < 0.0001; τ² = 3670.0922; I² = 98.85%) indicates that operative factors contribute to the observed variability.

Uniportal: The mean blood loss amount from uniportal procedures reached 68.44 mL with a standard error of 28.7 and a 95% CI between 12.18 and 124.71. The results showed statistical significance in Figure 7b and Table 12. The studies showed high levels of heterogeneity (Q(6) = 772.660, p < 0.0001; τ² = 5740.0004; I² = 99.76%).

**Table 12:** Random-effects meta-analysis and heterogeneity statistics for intraoperative blood loss in the uniportal approach Tau² estimator: restricted maximum-likelihood SE: standard error, CI: confidence interval, df: degrees of freedom

Parameter	Estimate	SE	Z	p value	95% CI		τ	τ² (SE)	I² (%)	H²	R²	df	Q	p (heterogeneity)
					Lower bound	Upper bound								
Intercept	68.4	28.7	2.38	0.017	12.175	124.708	75.763	5,740.0004 (3,330.7202)	99.76	416.808	-	6.000	772.660	-

Biportal vs. uniportal: The three comparative studies reported SMDs between -2.6422 and 0.2068, which showed that the uniportal approach performed better than the biportal approach in most cases (67%). The pooled SMD value was -0.9693 (95% CI = -2.6684 to 0.7298) (z = -1.1182, p = 0.2635) (see Figure 7c and Table 13), which did not reach statistical significance. The high level of heterogeneity (Q(2) = 179.6208, p < 0.0001; τ² = 2.2151; I² = 98.49%) persisted in the analysis. The study by Wang et al. showed potential outlier status, but no strong influence or publication bias was detected (p = 1.0000; p = 0.3042).

**Table 13:** Random-effects meta-analysis and heterogeneity statistics for intraoperative blood loss in uniportal vs. biportal approach Tau² estimator: restricted maximum-likelihood SE: standard error, CI: confidence interval, df: degrees of freedom

Parameter	Estimate	SE	Z	p value	95% CI		τ	τ² (SE)	I² (%)	H²	R²	df	Q	p (heterogeneity)
					Lower bound	Upper bound								
Intercept	-0.969	0.867	-1.12	0.263	-2.668	0.730	1.488	2.2151 (2.2546)	98.49	66.213	-	2.000	179.621	-

**Figure 8:**
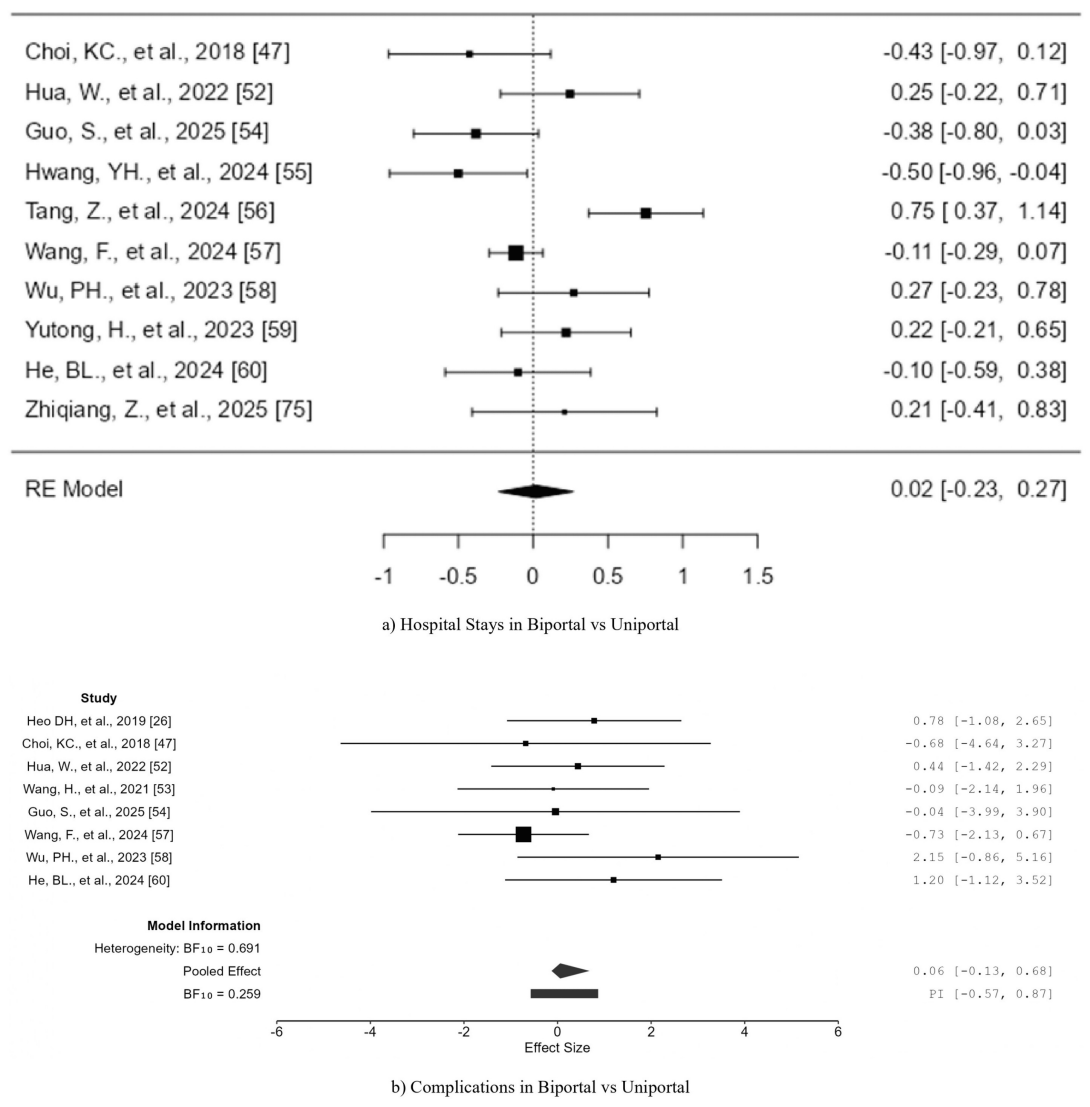
Forest plots comparing (a) hospital stays in biportal and uniportal approaches. (b) Complications in biportal and uniportal approaches RE: random effects

Hospital stay (biportal vs. uniportal): The postoperative hospital stay duration data came from ten studies, which included [47,52,54-60,75]. The SMD values spanned from −0.5009 to 0.7525, with equal numbers of positive and negative effects. The pooled SMD value of 0.0183 (95% CI = −0.2304 to 0.2670) did not reach statistical significance (z = 0.1443, p = 0.8853) (see Figure 8a and Table 14).

**Table 14:** Random-effects meta-analysis and heterogeneity statistics for hospital stay in uniportal vs. biportal approach Tau² estimator: restricted maximum-likelihood SE: standard error, CI: confidence interval, df: degrees of freedom

Parameter	Estimate	SE	Z	p value	95% CI		τ	τ² (SE)	I² (%)	H²	R²	df	Q	p (heterogeneity)
					Lower bound	Upper bound								
Intercept	0.0183	0.127	0.144	0.885	-0.230	0.267	0.330	0.1088 (0.0754)	72.33	3.615	-	9.000	30.303	-

The analysis showed moderate heterogeneity (Q(9) = 30.3035, p = 0.0004; τ² = 0.1088; I² = 72.33%) with a prediction interval spanning from -0.6744 to 0.7110. The study by Tang et al. showed potential outlier characteristics, but the analysis found no evidence of publication bias (p = 1.0000; p = 0.9994).

Complication (biportal vs. uniportal): Eight articles [19,47,52-54,57,58,60] reported complication rates in comparing uniportal with biportal endoscopy groups. A total of patients (uniportal endoscopy group 476 patients; biportal endoscopy group 460 cases) were included and summarized in Table 15. The Bayesian model yielded moderate evidence supporting the presence of heterogeneity among the included studies, with a posterior probability for heterogeneity of P(M|data) = 0.409 and an inclusion Bayes Factor (BF = 0.693). The evidence for a pooled overall effect was weaker (P(M|data) = 0.205, BF = 0.258), suggesting limited support for a consistent difference in complication rates across studies. The forest plot for the included study is shown in Figure 8b.

**Table 15:** Study-wise incidence of postoperative complications included in the meta-analysis

Study	Sample size (biportal/uniportal)	Number of complications (biportal/uniportal)								
		Dural tear	Incomplete decompression	Epidural hematoma	Nerve root injury	Iatrogenic instability	Transient dysesthesia	Infection	Lower limb numbness	Total complications
Heo et al. [26]	37/27	1/1	0/0	1/1	0/0	0/0	0/0	0/0	0/1	2/3
Choi et al. [47]	20/40	0/0	0/0	0/0	0/0	0/0	0/0	0/0	0/0	0/0
Hua et al. [52]	36/36	2/2	0/0	0/0	0/1	0/0	0/0	0/0	0/0	2/3
Wang and Wu [53]	23/25	1/1	0/0	0/0	0/0	0/0	1/1	0/0	0/0	2/2
Guo et al. [54]	44/46	0/0	0/0	0/0	0/0	0/0	0/0	0/0	0/0	0/0
Wang et al. [57]	235/240	2/2	0/0	0/0	3/1	0/0	0/0	1/0	0/0	6/3
Wu et al. [58]	32 / 29	0/3	0/0	0/0	0/0	0/0	0/0	0/0	0/0	0/3
He et al. [60]	33/32	0/1	0/0	0/0	0/0	0/0	1/2	0/0	0/0	1/3

The pooled effect estimate was 0.054 (95% CI = -0.160 to 0.686), with a 95% prediction interval ranging from -0.561 to 0.849. These results indicate that, on average, there was no statistically significant difference in the risk of postoperative complications between uniportal and biportal approaches, as the credible interval includes zero. The estimated between-study variance was τ² = 0.075 (95% CI = 0.000-0.655), corresponding to a heterogeneity estimate of τ = 0.137, suggesting low-to-moderate dispersion in true effect sizes across studies.

Under conditional modeling, which assumes the presence of heterogeneity, the pooled mean effect increased slightly to 0.266 (95% CI = -0.439 to 0.955), with an estimated τ² = 0.183 (95% CI = 0.004-1.104). The conditional 95% prediction interval (-0.592 to 1.190) suggests that future studies may report either a mild benefit or a small disadvantage for one technique over the other.

Overall, these findings demonstrate no significant difference in the incidence of postoperative complications between uniportal and biportal endoscopic surgeries, with modest heterogeneity observed among studies. The Bayesian evidence indicates that variability across studies is more likely driven by random differences rather than a consistent systematic effect favoring either surgical approach.

MacNab Score

A total of k = 6 studies comparing uniportal and biportal techniques were included in the analysis. Across individual studies, the proportion of patients achieving Excellent or Very Good outcomes on the MacNab scale varied, with ORs ranging from 0.17 to 1.71 (see Table 16). The pooled analysis using a model (DerSimonian-Laird method) yielded a combined log OR of -0.24 (SE = 0.40), corresponding to a pooled OR of 0.78, which suggests a trend favoring the biportal approach for excellent and very good MacNab outcomes, but the CI crosses 1 (0.36-1.70), so the difference is not statistically significant (p > 0.05). The studies show high heterogeneity, as I² equals 95%, indicating large differences between the research findings. The forest plot illustrates that individual study estimates were widely distributed, though most favored the Biportal approach.

**Table 16:** Comparative summary of reported MacNab score (event: excellent or very good) in uniportal vs. biportal approach OR: odds ratio of uniportal vs. biportal; values <1 favor biportal, >1 favor uniportal

Study	Uniportal (events/total)	Biportal (events/total)	OR	logOR	var(logOR)
Hua et al. [52]	33/36	34/36	0.647	-0.435	0.8930
Guo et al. [54]	36/46	42/44	0.171	-1.764	0.6516
Tang et al. [56]	48/52	58/61	0.621	-0.477	0.6214
Wang et al. [57]	212/240	192/235	1.696	0.528	0.0689
Zhiqiang et al. [75]	23/25	16/17	0.719	-0.330	1.6060
He et al. [60]	29/32	30/33	0.967	-0.034	0.7345

Discussion

The present study assessed and compared the clinical findings associated with uniportal and biportal endoscopic spine surgery across 53 studies with a variety of patient populations and procedural variations. The research shows that both methods produce substantial improvements in functional recovery and pain management through their ability to reduce ODI and VAS scores for back and leg pain. The pooled estimates showed a preference for the biportal technique, although effect sizes remained small and consistent between groups.

Pain and Functional Outcomes

The postoperative results for all studied parameters, including VAS for leg and back pain and ODI, showed significant improvement from baseline for both uniportal and biportal endoscopic techniques. The SMD values for VAS (leg pain) measurements exceeded 5 in both groups, which shows substantial and clinically important effects. The study results validate earlier systematic reviews, which showed that endoscopic decompression through minimally invasive techniques reduces neural compression symptoms while improving functional results.

The two methods showed a small difference in their combined SMD values, which supported the biportal technique. The biportal technique provides superior visualization and better control through its independent viewing and working channels, which explains this observed difference. The combination of specialized endoscopic and surgical instruments allows for improved bleeding control and accelerated decompression methods when treating complex or multiple-level cases. The biportal setup benefits from continuous saline irrigation because it produces a clearer surgical field and minimizes thermal tissue damage, which results in better postoperative results.

Operative Time, Blood Loss, and Hospital Stay

The results showed that uniportal and biportal surgical approaches produce equivalent results in both surgical execution and postoperative healing because they produce equivalent operative times and intraoperative blood loss. The studies showed varying operative times because surgeons' experience levels and case difficulty, as well as procedural consistency standards, differed.

Theoretical advantages of biportal surgery include quick decompression times for experienced surgeons, but the complex nature of the procedure may extend the time before these benefits become available. The small differences in surgical blood loss between biportal (64.61 mL) and uniportal (68.44 mL) procedures do not affect patient outcomes because endoscopic procedures already result in minimal blood loss. The hospital stay duration between these two groups was equivalent because both methods enabled patients to initiate movement at times that aligned with minimally invasive surgical protocols.

MacNab Score and Complications

The pooled analysis of MacNab outcomes showed no significant difference between the two techniques (pooled OR = 0.78; 95% CI = 0.36-1.70) because both uniportal and biportal approaches resulted in similar functional recovery results. The biportal technique received backing from particular studies, yet the CI exceeded unity, which shows the reported advantage failed to achieve statistical significance. The high between-study heterogeneity (I² ≈ 95%) indicates significant differences between studies because of their varying research approaches, participant numbers, observation periods, and surgical practitioner qualifications. The research shows that both methods produce positive results for patients when they receive suitable selection.

Heterogeneity and Bias Considerations

The high heterogeneity (I² > 95%) observed in most analyses stems from differences between studies because different clinical and methodological approaches were used in the studies. The combination of different surgical approaches, patient selection, outcome measurements, follow-up duration, and different study designs (retrospective and prospective) leads to different effect size results regarding their designs and indications for surgery and follow-up periods, and the surgeon's experience levels. The various surgical methods used in pooled analyses become more evident because endoscopic spine surgery outcome reports do not follow standardized reporting protocols.

The research indicates that scientists choose to release findings that prove new methods produce beneficial results through different assessment criteria. The sensitivity analyses produced results that showed the same direction of effects, which validated the stability of the main research findings.

The Bayesian meta-analysis of postoperative complications showed no important distinction between the two surgical methods. The posterior probability of a significant combined effect was small (P(M|data) = 0.205) while the 95% credible interval for the combined effect (-0.160 to 0.686) included the null value. The results from conditional modeling with heterogeneity assumption were similar to the previous results (pooled effect = 0.266; 95% CI = -0.439 to 0.955), which confirmed that the complication rates were not statistically or clinically different. The between-study variance (τ² ≈ 0.075-0.183) showed a small to moderate level of heterogeneity, which suggested that the different complication profiles in the studies did not affect the general safety assessment.

Collectively, these findings suggest that both uniportal and biportal endoscopic techniques provide similar safety and functional outcomes. The biportal method provides better visualization and instrument access during surgery, but the uniportal method keeps the benefits of minimal tissue damage and fast procedure duration.

Clinical Implications

The results of this meta-analysis support the use of both uniportal and biportal endoscopic techniques as effective, minimally invasive alternatives to conventional open or microscopic decompression. The slightly superior functional and pain outcomes with the biportal approach may justify its use in patients with more extensive pathology or where wider decompression is required. Conversely, the uniportal approach remains a valuable technique, particularly in single-level disease, due to its simplicity, smaller incision, and reduced instrumentation requirements.

**Figure 9:**
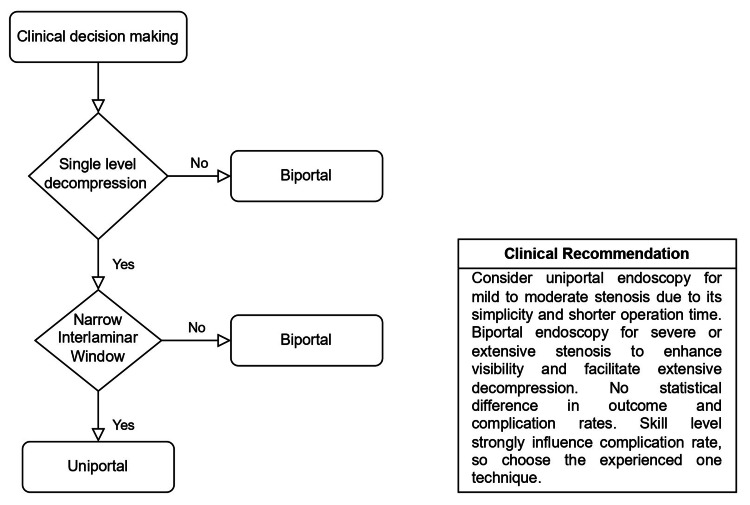
Decision-making framework comparing uniportal and biportal endoscopic lumbar spine surgery, including procedural selection pathway and evidence-based clinical recommendations Image credit: This is an original image created by the authors Purushottam Kumar and Suyash Singh

Importantly, the findings highlight the role of surgeon experience and case selection in determining outcomes. Standardized training, procedural protocols, and long-term comparative trials are essential to establish evidence-based selection criteria for each approach. In line with our findings, we have recommended a clinical decision-making framework (see Figure 9).

Limitations

The majority of included studies used retrospective designs while showing different research approaches. The available subgroup analyses for spinal level and pathology type, and surgeon experience were restricted because of inconsistent reporting practices. The pooled estimates may have become exaggerated because of publication bias and small-study effects. The observed trends gain additional validity from the large combined sample size and the consistent pattern of results despite these study restrictions.

Future Directions

Future studies need to perform high-quality multicenter RCTs focusing on high-priority subgroups such as patients with multilevel stenosis, recurrent disc herniation, large migrated herniations, elderly patients, and those with mixed degenerative pathologies, that compare these two techniques using standardized outcome measures and follow patients for an extended duration. The clinical decision process would benefit from additional studies about learning curve measurement and cost-effectiveness analysis, and patient-reported quality-of-life assessment. The combination of image-guided navigation with robotic assistance has the potential to enhance both precision and safety, which could minimize the performance difference between the two endoscopic techniques.

## Conclusions

Both uniportal and biportal endoscopic spine surgeries are safe and effective minimally invasive techniques for the management of LDD. The biportal technique shows a small but steady advantage in clinical performance when compared to the other two approaches which both lead to better pain and functional results. The selection between these techniques should focus on surgeon experience and case difficulty and hospital equipment availability because the procedures show no substantial variations in surgical duration or blood loss or hospital stay.
